# Multi-peptide ELISAs overcome cross-reactivity and inadequate sensitivity of conventional *Chlamydia pneumoniae* serology

**DOI:** 10.1038/s41598-019-51501-5

**Published:** 2019-10-21

**Authors:** Kh Shamsur Rahman, Bernhard Kaltenboeck

**Affiliations:** Department of Pathobiology, College of Veterinary Medicine, Auburn University, Auburn, AL USA

**Keywords:** Infectious-disease diagnostics, Bacterial infection

## Abstract

Cross-reactivity of classical chlamydial antigens compromises *Chlamydia (C*.*) pneumoniae* serology. By testing with 185 human antisera, we expanded 18 previously discovered *C*. *pneumoniae*-specific B-cell epitopes to 48 peptide antigens from 12 *C*. *pneumoniae* immunodominant proteins. For specific detection of antibodies against *C*. *pneumoniae*, we developed novel ELISAs with strongly reactive individual peptide antigens and mixtures of these peptides. By comparison to a composite reference standard (CRS) for anti-*C*. *pneumoniae* antibody status of human sera, the top-performing CpnMixF12 peptide assay showed 91% sensitivity at 95% specificity, significantly higher than 4 commercial anti-*C*. *pneumoniae* IgG ELISAs (36-12% sensitivity at 95% specificity). Human *C*. *pneumoniae* (Cpn) and *C*. *trachomatis* (Ctr) seroreactivity was 54% biased towards co-positivity in commercial Cpn and Ctr ELISAs, but unbiased in Cpn and Ctr peptide antibody assays, suggesting severe cross-reactivity of commercial ELISAs. Using hyperimmune mouse sera against each of 11 *Chlamydia* spp., we confirm that commercial Cpn and Ctr ELISA antigens are cross-reactive among all *Chlamydia* spp., but Cpn and Ctr peptide antigens react only with antisera against the cognate chlamydial species. With simultaneously high specificity and sensitivity, and convenient use for non-specialized laboratories, these ELISAs have the potential to improve serodiagnosis of *C*. *pneumoniae* infection.

## Introduction

Intracellular *Chlamydia* (*C*.) spp. bacteria infect virtually all vertebrates and cause largely chronic and asymptomatic diseases. The principal human chlamydial pathogens are *C*. *pneumoniae* and *C*. *trachomatis*^1–4^. The single human serovar of *C*. *pneumoniae* is a common cause of respiratory infection, leading to pharyngitis, bronchitis, and community-acquired pneumonia^1,2^. *C*. *trachomatis* serovars cause ocular and sexually transmitted genitourinary tract infections, and lymphogranuloma venereum^3,4^. *C*. *psittaci* sporadically causes severe zoonotic pneumonia^5,6^.

Most respiratory *C*. *pneumoniae* infections are mild or asymptomatic^7,8^, similar to *Mycoplasma* infections, although severe pneumonia can develop in elderly patients and those with coexisting cardiopulmonary diseases^9,10^. Infection with *C*. *pneumoniae* occurs worldwide, resulting in 40–90% prevalence of serum antibodies to classical *C*. *pneumoniae* antigens^11–14^. *C*. *pneumoniae* has been associated with both epidemic and endemic occurrences of acute respiratory disease, and with 6–20% of all community-acquired pneumonias and 5% of bronchitis and sinusitis cases in adults and children^9,10,15–18^.

Diagnosis of *C*. *pneumoniae* infection is preferably based on the isolation of the organism or its detection by PCR, the preferred method of diagnostic testing recommended by CDC for acute *C*. *pneumoniae* infection^2^. However, appropriate specimens require invasive sampling, and for that reason serology is currently the convenient tool most often applied for the routine diagnosis of *C*. *pneumoniae* infections^2,19–24^. In addition, serological assays indicate the history of exposure to *C*. *pneumoniae* and are preferable over antigen detection for epidemiologic or retrospective analyses.

Available serological tests for detection of anti-*C*. *pneumoniae* antibodies include enzyme-linked immunosorbent assays (ELISA) and the micro-immuno-fluorescence (MIF) test^25–30^. The high prevalence of *C*. *trachomatis* infection^28–30^ complicates results of testing for *C*. *pneumoniae* antibodies due to the possibility of false seropositivity arising from *C*. *trachomatis* infections^31–40^. ELISAs based on *C*. *pneumoniae* elementary bodies (EB) or outer membrane complex (OMC) suffer from lack of specificity due to cross-reactivity of *Chlamydia* genus-specific antigens. Similarly, the majority of immundominant protein candidate antigens for anti-*C*. *pneumoniae* ELISAs (OmpA, Omp2, PorB, or Hsp60) is highly conserved within *Chlamydia* spp.^2,32,33,39,40^, and thus poorly suited for *C*. *pneumoniae*-specific ELISAs. The MIF test was initially developed for species/serovar-specific detection of anti-*C*. *trachomatis* antibodies^25–27^, and later adopted for *C*. *pneumoniae* serology^9–15^. The MIF test has remained the gold standard in *C*. *pneumoniae* serological testing because of higher specificity and sensitivity than ELISAs^16,19^.

Purified EBs, the antigenically complex infective forms of *Chlamydia*, are used as MIF antigens^25–27^. Outer membrane protein A (OmpA), the serovar-determining most immunodominant protein of *C*. *trachomatis*, is the main constituent antigen of the MIF EB antigens. For *C*. *trachomatis* MIF serology, the OmpA antigen produces strong reactivity with anti-*C*. *trachomatis* antibodies during microscopic observation of MIF slides. Consequently, skilled personnel can identify a pattern of specific versus non-specific reactivity in the *C*. *trachomatis* MIF test. However, this microscopic observation is a painstaking technique, requiring extensive expertise and subjective interpretation of EB reactivity with anti-*Chlamydia* spp. antibodies, imposing a risk of high inter-laboratory variation in results^19^. Nevertheless, the *C*. *trachomatis* serovar EB antigens can still provide a good degree of species- and serovar-specificity in the MIF test.

Several studies suggest that the *C*. *pneumoniae* MIF test is less sensitive and specific than its general perception^2,22,23,36,37^. For *C*. *pneumoniae* serology, the MIF test is problematic due to the much lower immunogenicity of the *C*. *pneumoniae* OmpA antigen^32^. For example, the *C*. *pneumoniae* MIF test failed to detect anti-*C*. *pneumoniae* antibodies from sera of *C*. *pneumoniae* PCR/culture-positive children, underscoring the poor sensitivity of the *C*. *pneumoniae* MIF test^2,7,8,22^. Additionally, *C*. *pneumoniae*, *C*. *trachomatis*, and *C*. *psittaci* EB MIF antigens detected anti-*C*. *pneumoniae* antibodies without marked difference in the MIF antibody titers^36,37^. This serious cross-reactivity and poor sensitivity of the *C*. *pneumoniae* MIF test, together with cumbersome procedures inherently associated with the MIF technique and high inter-laboratory variation in MIF titers, stress the need to identify *C*. *pneumoniae*-specific antigens^2,7,20–24^. Therefore, development of novel specific and sensitive assays, particularly in simple format such as enzyme-linked immunosorbent assay (ELISA), is urgently needed for human chlamydial serology.

Previously, we have identified highly reactive and specific B cell epitopes^41–47^ of immunodominant proteins of all *Chlamydia* species^48–51^. In extensive evaluation^46,47^, we also showed that the *C*. *trachomatis*-specific peptide antigens provide superior assays with high sensitivity and specificity. Importantly, the high sensitivity of the peptide assays was mainly achieved by the use of multiple B-cell epitopes of several *C*. *trachomatis* immunodominant proteins^46,47^. Since antibody responses to individual B cell epitopes are stochastic^41–46^, only the combined use of multiple peptide antigens reliably measured host antibodies produced in response to *C*. *trachomatis* infection^46^, similar to the quantitative results obtained with complex antigens. In the present study, we developed and validated peptide assays for detection of anti-*C*. *pneumoniae* antibodies. Starting from 18 previously identified peptides^41,45^, we expanded the repertoire to 48 human sero-reactive *C*. *pneumoniae* peptide antigens by testing with human sera. Using optimal subsets of these 48 peptide antigens, we established simple, yet highly specific and sensitive peptide ELISAs for detection of anti-*C*. *pneumoniae* antibodies.

## Results

### Reactivities of *C*. *pneumoniae*-specific peptide antigens with human serum pools

For identification of *C*. *pneumoniae*-specific peptide antigens, a panel of 153 *C*. *pneumoniae* peptide antigens with high predicted score for B-cell epitopes^41–43^ was initially tested with 4 human serum sub-pools of 185 donors. With combined signal intensities (average of 4 OD values) with the 4 human serum pools, all 153 *C*. *pneumoniae* peptides were initially ranked and a set of 48 top ranked peptides from 12 *C*. *pneumoniae* immunodominant proteins was selected for further evaluation (Table 1). Peptide antigens with the highest-ranked-reactivities were identified from *C*. *pneumoniae* IncA/IncCT119, followed by Pmp6G/I, Pmp21D, OmpA/MOMP, YopC/GspD, CT618/IncCT618, Pmp11G/I, CT529/IncCT529, Pmp2G/I, YwbM/CPn0677, CrpA/CT442, and PdhC (Table 1 and see Supplementary Table S1).

**Table 1:** *Chlamydia pneumoniae*-specific peptide antigens.

Protein^a^	Peptide # ^b^	Peptide (Position, AA#)^c^	Peptide sequence^d^	Human serum reactivity^e^		Mouse serum reactivity (RLU)^f^			Human host-dependent^g^	Sequence conservation (%, identity)^h^		
				RLU	Rank #	Cpn	Ctr	Cps		Cpn	Ctr	Chl
IncA/IncCT119	1	331–370	QKAESEFIACVRDRTFGRRETPPPTTPVVEGDESQEEDEG	7,062	1	132,193	0	0	No	83–100	≤40	≤40
	2	331–345	QKAESEFIACVRDRT	4,356	3	6,372	0	0	No	80–100	≤40	≤40
	3	336–350	EFIACVRDRTFGRRE	2,565	4	26,483	0	0	No	93–100	≤40	≤40
	4	344–363	RTFGRRETPPPTTPVVEGDE	1,441	11	49,698	0	0	No	86–100	≤40	≤40
	5	361–375	GDESQEEDEGGTPPV	623	30	11,520	0	0	No	93–100	≤40	≤40
Pmp6G/I	6	222–237	TATDKGGGIYSKEKDS	5,172	2	0	0	4	Yes	94–100	44	44–50
	7	947–962	AGTTLETTTTNNTDGS	1,649	8	0	0	0	Yes	100	≤40	≤40
	8	181–196	AKTTTAALLDQNTSTK	1,640	9	0	0	0	Yes	100	≤40	40–47
	9	164–179	NTSEKDGAAVSAYSID	1,402	12	0	0	0	Yes	100	≤40	40–56
	10	1223–38	HGQVSYGRNHHNMTTK	1,366	13	0	0	0	Yes	100	≤40	40–50
	11	899–914	QDASIPANTTTILNQK	895	18	0	0	0	Yes	100	≤40	40–44
	12	872–887	ASVPVVPVAPANPNTG	813	21	0	0	0	Yes	100	≤40	≤40
	13	697–712	DIATKSLTLTENESLS	776	27	0	0	0	Yes	100	≤40	≤40
	14	1032–47	NLDDFNPIPSSMAAPD	529	36	0	0	0	Yes	100	≤40	≤40
	15	714–729	INNTAKRSGGGIYAPK	526	37	0	0	0	Yes	100	≤40	≤40
Pmp21D	16	1119–34	SQVDSSAPLPTENKEE	1,841	5	0	0	0	Yes	100	≤40	≤40
	17	140–155	DTSNAVSEKISSDTKE	1,540	10	0	0	0	Yes	100	≤40	≤40
	18	640–655	TAPVESDASSTNKDEK	1,356	14	0	0	0	Yes	100	≤40	≤40
	19	191–206	EDLEISENISARDPLQ	1,202	15	0	0	0	Yes	100	≤40	≤40
	20	147–186	EKISSDTKENRKDLETEDPSKKSGLKEVSSDLPKSPETAV	988	17	62,389	4	0	No	100	≤40	≤40
	21	1131–70	NKEETLVSAGVQINMSSPTPNKDKAVDTPVLADIISITVD	561	32	53,127	0	0	No	100	≤40	≤40
	22	654–693	EKSLNACSHGDHYPPKTVEEEVPPSLLEEHPVVSSTDIRG	542	33	63,913	0	0	No	100	≤40	≤40
	23	717–732	LGGGEESSTVGDLAIV	375	42	0	0	0	Yes	100	≤40	≤40
	24	1121–36	VDSSAPLPTENKEETL	306	44	0	0	0	Yes	100	≤40	≤40
	25	521–536	RSNPKLEQKDSGENIN	298	45	0	0	0	Yes	100	≤40	≤40
OmpA/MOMP	26	242–257	VAFPLPTDAGVATATG	1,061	16	7,549	0	0	No	100	≤40	40–63
	27	158–173	FGVKGTTVNANELPNV	804	23	6,013	0	0	No	88–100	≤40	40–63
	28	309–324	AVLNLTAWNPSLLGNA	292	46	51,609	826	836	No	94–100	≤40	40–81
	29	089–104	PTGSAAANYTTAVDRP	77	47	48,711	0	0	No	94–100	≤40	40–75
YopC/GspD	30	077–092	HTKKTTPGSIPSKVFS	826	20	0	0	0	Yes	100	≤40	≤40
	31	104–119	KTSGSAFPAKPTTLKE	391	40	47	0	0	Yes	100	≤40	≤40
	32	196–211	TEKDVQPKTQATPHAS	381	41	0	0	0	Yes	94–100	≤40	≤40
CT618/IncCT618	33	201–216	PETISDPENRNKPSAE	811	22	76,037	0	0	No	100	≤40	≤40
Pmp11G/I	34	487–502	GLKQPVSLTAKGASNK	795	25	0	0	0	Yes	100	≤40	40–44
	35	720–735	GRAKFSESAIEKFPRE	778	26	0	0	0	Yes	100	≤40	≤40
	36	888–903	LLRGSNNYVYNSNCEL	771	28	0	0	0	Yes	100	≤40	40–44
	37	334–349	FVRNTLTTTGSTDTPK	446	39	0	0	0	Yes	100	≤40	≤40
CT529/IncCT529	38	236–275	RAKESLYNERCALENQQSQLSGDVILSAERALRKEHVATL	1,816	6	5,741	0	0	No	100	≤40	≤40
	39	139–254	KERKTPGEYSKMLLTR	1,773	7	6	0	0	Yes	94–100	≤40	≤40
Pmp2G/I	40	064–079	VVIENVPKTGETQSTS	833	19	15	0	0	Yes	100	≤40	≤40
	41	024–039	AATTEELSASNSFDGT	447	38	0	0	0	Yes	100	≤40	40–69
YwbM/CPn0677	42	265–280	DPGESIQNFLETRVSD	801	24	0	0	0	Yes	100	≤40	≤40
	43	209–244	TSSTGPVPQAVTVAKD	673	29	21	0	0	Yes	100	≤40	≤40
	44	233–248	AIKNLENPKPGNDPDG	535	35	0	0	0	Yes	94–100	≤40	≤40
CrpA/CT442	45	173–188	TTPVLNDGRGTPVLSP	581	31	72	0	0	Yes	71–100	≤40	≤40
	46	181–196	RGTPVLSPLVSKIARV	29	48	0	0	0	Yes	94–100	≤40	≤40
PdhC	47	91–106	PKTEPSNLEASPKGSS	541	34	95	0	0	Yes	100	≤40	≤40
	48	105–120	SSEEVSPATTPQAASA	346	43	105	0	0	Yes	100	≤40	≤40

^a^From 12 immunodominant proteins of *C*. *pneumoniae* strain CWL029, a set of 48 *C*. *pneumoniae*-specific peptide antigens with highest reactivity is shown.

^b^These 48*C*. *pneumoniae*-specific peptide antigens are numbered (#1 to #48) for ease in peptide antigen preparation and reporting composition of Cpn peptide antigen mixes.

^c^The amino acid (AA) positions of the peptide sequence in each full-length protein of *C*. *pneumoniae* strain CWL029 is shown.

^d^Only the actual chlamydial sequence of peptide antigens is shown, without N-terminal biotin and serine-glycine-serine-glycine (SGSG) spacer that is attached to each peptide.

^e^Average peptide antigen reactivity in relative light units (RLU) in chemiluminescent ELISA by use of 4 sub-pooled human sera of 185 blood donors is shown.

^f^Peptide antigen reactivity with hyperimmune mouse sera pooled from 16–50 mice is shown. Cpn indicates *C*. *pneumoniae*-specific pooled sera that were raised in mice by 3 × intranasal inoculation of live *C*. *pneumoniae* CWL029 bacteria as previously described^41,45^. Ctr indicates *C*. *trachomatis* D/UW-3/CX; and Cps, *C*. *psittaci* 02DC15.

^g^‘No’ indicates that the peptide antigen is recognized by both human and mouse pooled sera. Yes, indicates that the antigen is recognized by human sera, but not or only minimally recognized by mouse antisera.

^h^Cpn indicates range of sequence conservation within 6 strains of *C*. *pneumoniae* (CWL029, TW-183, AR39, J138, B21, and LPCoLN). Ctr indicates sequence conservation between *C*. *pneumoniae* CWL029 and *C*. *trachomatis* D/UW-3/CX, and Chl for sequence conservation between *C*. *pneumoniae* CWL029 and each of the remaining 9 *Chlamydia* spp. Sequence identities of 45%, 60%, 75%, and 90% translate into peptide cross-reactivity probabilities of 0.02, 0.12, 0.46, and 0.84, respectively^41^. Sequences with identities below 40% typically cannot be aligned correctly and the probability of peptide cross-reactivity is less than 1%^41^.

In confirming these 48 Cpn peptide antigens with mouse anti-*C*. *pneumoniae* antisera^41,45^, only a limited set of 14 *C*. *pneumoniae* peptides from 5 proteins (IncA, Pmp21D, OmpA, CT618, and CT529; Table 1) showed strong reactivities. Overall, the natural human-hosts of *C*. *pneumoniae* produced antibody responses against a wide range of proteins as well as multiple protein regions of single proteins (Table 1), compared to the much more limited response of the non-natural murine-host (Table 1). This finding is in agreement with our previous report that human-hosts produce antibody responses against wide-spectrum proteins of *C*. *trachomatis* than murine-hosts^45,46^.

### Sequence conservation of the Cpn B-cell epitopes within *Chlamydia* spp. and *C*. *pneumoniae* strains

The majority of these 48 peptide antigens, except for CpnOmpA (≤81% sequence identity), are highly divergent from other *Chlamydia* spp. (≤40% identity, Table 1), indicating *C*. *pneumoniae* specificity^41,43^. Importantly, anti-*C*. *trachomatis* or *C*. *psittaci* antisera did not cross-react with these peptide antigens, with exception of CpnOmpA_309–324 peptide that produced very low cross-reactivities with mouse anti-*C*. *trachomatis* and *C*. *psittaci* antisera (Table 1). Additionally, these peptide antigen sequences were highly conserved within 6 strains of *C*. *pneumoniae* (94–100% sequence identity among CWL029, TW-183, AR39, J138, B21, and LPCoLN; Table 1), with exception of the CpnCrpA (≥71% identity), IncA (≥80%), and OmpA (≥88%). These results indicate that a combination of these Cpn peptide antigens will detect host antibody responses against any of the *C*. *pneumoniae* strains.

### Specificity confirmation of *C*. *pneumoniae* peptide mixes

In an approach to simplify serological testing by use of mixed rather than multiple individual peptide antigens, we tested 13 mixes of subsets of the 48 Cpn peptides (5–48 peptides/mix; see Supplementary Fig. S2) with pools of anti-*Chlamydia* mouse sera and confirmed highly specific reactivities (see Supplementary Table S3). Out of 13 Cpn peptide mixes, 12 mixes were reactive with mouse anti-*C*. *pneumoniae* antisera, and none of them showed cross-reactivity with anti-*C*. *trachomatis* and anti-*C*. *psittaci* antisera (Table S3). Only one mix (CpnMix B10) was completely non-reactive with anti-*C*. *pneumoniae* mouse antisera (Table S3), which was expected given that all of the component Pmp6G/I peptides were not reactive with mouse anti-*C*. *pneumoniae* sera (Table 1). As expected, the reactivity magnitudes of these Cpn mixes were largely influenced by the number and signal strength of individual component peptide antigens that were reactive with mouse anti-*C*. *pneumoniae* sera (Tables 1, S3). Overall, the Cpn peptide mixes showed high specificity similar to single peptide antigens, albeit the signal magnitude of mixed peptide assays was lower than that of the strongest component individual peptides, analogous to the earlier reported reactivities of *C*. *trachomatis* peptide mixes^47^.

**Anti-*C***. ***pneumoniae*** **IgG antibodies detected by 29 different assays**

**Figure 1:**
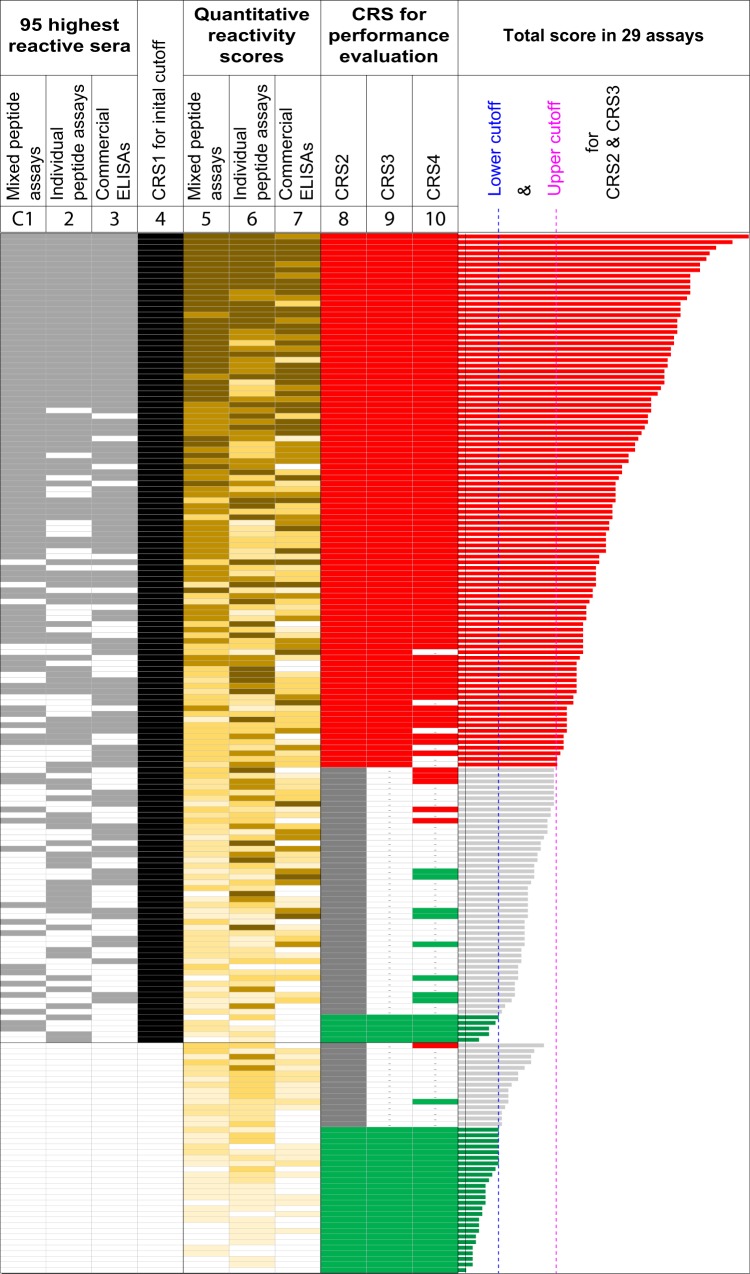
Anti-*C*. *pneumoniae* IgG antibodies detected by 29 assays. To identify sets of anti-*C*. *pneumoniae* antibody-positive and -negative sera, a panel of 185 blood donor sera was analyzed with 13 mixed and 12 individual Cpn peptide antigen assays, and 4 commercial *C*. *pneumoniae* ELISAs (details shown in Fig. S4). Analyses in the first three columns (C1-C3) are input for the preliminary composite reference standard 1 (CRS1) in C4. For each serum, the sum of the squared OD values of the individual assays in each category was determined. Grey indicates antibody positivity among the 95 highest reactive sera in combined scoring of the assay categories. Cutoff-independently, the 95 top-ranked reactive sera were specified as positive for anti-*C*. *pneumoniae* antibodies, matching the mean 51.4% anti-*C*. *pneumoniae* IgG antibody prevalence in the study population determined by 4 commercial ELISAs. Using a 80.5% specificity cutoff based on CRS1, quantitative reactivity scores for assay categories C5-C7 were determined. These scores were derived from the sum of quartile positive reactivities of all component assays of each category (Fig. S4). The total score of all 29 individual assays for each serum, shown in the right bar graph, was derived from the sum of all individual assay scores. For assay performance evaluation, CRS2, CRS3, and CRS4 (C8-C10) were derived. For CRS2, 154 sera with the highest combined score in the 29 assays were considered antibody-positive (red & grey), and 31 sera with the lowest scores were considered antibody-negative (green). CRS3 was derived from CRS2 after exclusion of 59 borderline -positive sera (grey). CRS4 was constructed as peptide assay-based standard from the combined scores of 13 mixed and 12 individual peptide assays (without the 4 commercial ELISAs). The 95 sera with the highest score were considered antibody-positive (red), 40 sera with the lowest scores were considered antibody-negative (green), and 50 weakly-positive sera were excluded.

To identify anti-*C*. *pneumoniae* antibody-positive and -negative sera, a panel of 185 human blood donor sera was tested with *C*. *pneumoniae* IgG antibody detection assays of three categories (Fig. 1, and see Supplementary Fig. S4): (i) 13 Cpn peptide mixes prepared by mixing 5–48 Cpn peptide antigens (A5-M48; Fig. S2, Table S3); (ii) 12 individual strongly reactive Cpn peptide antigens from 8 *C*. *pneumoniae* proteins (Table 1, Fig. S4); and (iii) 4 commercial *C*. *pneumoniae* IgG antibody ELISAs based on (A) Cpn EB antigen (Savyon), (B) Cpn OMC antigen (Serion), (C) lysate antigen of cell-culture propagated Cpn (Euroimmun), and (D) chlamydial LPS-free proprietary purified Cpn antigen (Medac). In the absence of a definitive standard for anti-*C*. *pneumoniae* antibodies, the initial composite reference standard 1 (CRS1) was constructed from the sum of the squared OD values of each serum (Figs 1. S4). The equally weighted consensus of the three assay categories resulted in 144 positive and 41 negative sera (77.8% anti-*C*. *pneumoniae* antibody prevalence; Figs 1, S4).

### Average assay sensitivities relative to composite reference standards for human anti-*C*. *pneumoniae* IgG status

Two composite reference standards (CRS2 and CRS3) derived from the 29 individual assays were used to evaluate assay performances. CRS2 was constructed from the total score in the 29 component assays (Figs 1, S4), resulting in 154 positive and 31 negative sera for anti-*C*. *pneumoniae* antibodies (83.2% prevalence). At 98%, 95%, 90%, 85%, and 80% specificity cutoffs, Cpn Mix F12 achieved the highest average sensitivity (74%), followed by Cpn Mix K24 and J20 (73–72% sensitivity; Table 2). Compared to the mixed peptide assays, sensitivities with single peptide antigens were lower, with 55–21% average sensitivities for the 8 top-ranked individual peptides (Table 2). Combining the results of the 8 peptides substantially improved assay performance to an average of 72% sensitivity at the same 98–80% specificity cutoffs (Table 2). Thus, the commercial ELISAs achieved lower sensitivity (56-68% sensitivity; Table 2) than top-performing Cpn Mixes (F12, K24, or J20) or the combined 8 individual peptide antigens.

**Table 2:** Assay sensitivities for detection of human anti-*C*. *pneumoniae* IgG^a^.

Assay type		Rank^#^	Antigen	Average sensitivity^b^, %	
				CRS2 (154 Pos vs 31 Neg)^c^	CRS3 (95 Pos vs 31 Neg)^d^
Cpn mixed peptides	Single Mix	1	Cpn Mix F12	74	97
		2	Cpn Mix K24	73	91
		3	Cpn Mix J20	72	90
		4	Cpn Mix M48	69	88
		5	Cpn Mix G12	68	88
		6	Cpn Mix A5	63	85
		7	Cpn Mix D12	60	80
	Combined^e^	—	A5 + D12 (Average OD)	71	93
Cpn individual peptides	Single peptide	1	Cpn_IncA_331–370	55	74
		2	Cpn_OmpA_158–173	37	45
		3	Cpn_Pmp21D_1131–170	33	39
		4	Cpn_CT618_201–216	31	38
		5	Cpn_Pmp6G/I_181–196	34	36
		6	Cpn_OmpA_089–104	29	33
		7	Cpn_YopC_077–092	23	28
		8	Cpn_Pmp21D_0147–86	21	25
	Combined^f^	1	Avg OD of 6 Pept (#1–6)	72	85
		2	Avg OD of 8 Pept (#1–8)	70	84
		3	Avg OD of 4 Pept (#1–4)	64	80
Cpn ELISAs	Medac	1	Proprietary	67	84
	Serion	2	Cpn OMC	68	82
	Savyon	3	Cpn EB	66	81
	Euroimmun	4	Cpn lysates	56	74

^a^Assay sensitivities for each test were determined from receiver operating characteristic (ROC) curves. The binomial anti-*C*. *pneumoniae* IgG antibody status was used as *X* categorical variable known *a priori*, and serum reactivities were used as *Y* continuous predictor variable (Figs 1, S4).

^b^The average assay sensitivity (%) was calculated from the mean of 5 different sensitivities obtained at 98%, 95%, 90%, 85% and 80% specificities.

^c^Composite reference standard 2 (CRS2) for binomial anti-*C*. *pneumoniae* antibody status was derived from the total reactivity score of 29 individual tests (*C*. *pneumoniae*-specific 13 peptide mixes, 12 single peptide antigens, and 4 commercially available *C*. *pneumoniae* antibody ELISAs; Figs 1, S4). Of all 185 human sera, 154 sera with the highest combined score were considered positive, and 31 sera with the lowest score were considered negative for anti-*C*. *pneumoniae* IgG antibodies (Figs 1, S4).

^d^CRS3 was derived after exclusion of 59 weakly positive sera (borderline sera shown with grey-bar in Figs 1, S4) out of the 185 sera. A total of 95 sera with the highest combined score was considered positive, a total of 31 sera with the lowest score were considered negative (126 sera in total; Figs 1, S4).

^e^This new combined test was derived by averaging two OD values of 2 separate tests with Cpn peptide mixes (Cpn Mix A5 and D12; Fig. S2, Table S3).

^f^Combined tests for single peptide assays are derived by averaging OD values of 4, 6, or 8 separate tests with single Cpn peptides.

In CRS2 evaluation, the performance of peptide assays for anti-*C*. *pneumoniae* antibodies was lower than those of the previously reported *C*. *trachomatis* peptide antigen assays^46^. This discrepancy may be due to poorly defined antibody positivity in CRS2 that has a high 83.2% prevalence of anti-*C*. *pneumoniae* IgG (Figs 1, S4). In the commercial ELISAs, we determined in average a 51.4% anti-*C*. *pneumoniae* IgG prevalence. This translates into 95 positives out of 185 sera. To improve the quality of the reference standard CRS2, we constructed CRS3 from the 95 strongest reactive sera (positive) and the same set of 31 weakest reactive (negative) sera, but excluded ambiguous 59 weakly reactive sera (borderline) that were included in CRS2 (Figs 1, S4). Comparison of average sensitivities demonstrated that relative to CRS3 all assays performed substantially better than to CRS2 (Table 2). This suggests that CRS3 evaluation may represent a realistic epidemiological situation in which individuals with high anti-*C*. *pneumoniae* antibody levels are contrasted with individuals with baseline serum reactivity (antibody-negative).

### Distribution of anti-*C*. *trachomatis* antibodies in anti-*C*. *pneumoniae* antibody-positive and -negative sera

**Figure 2:**
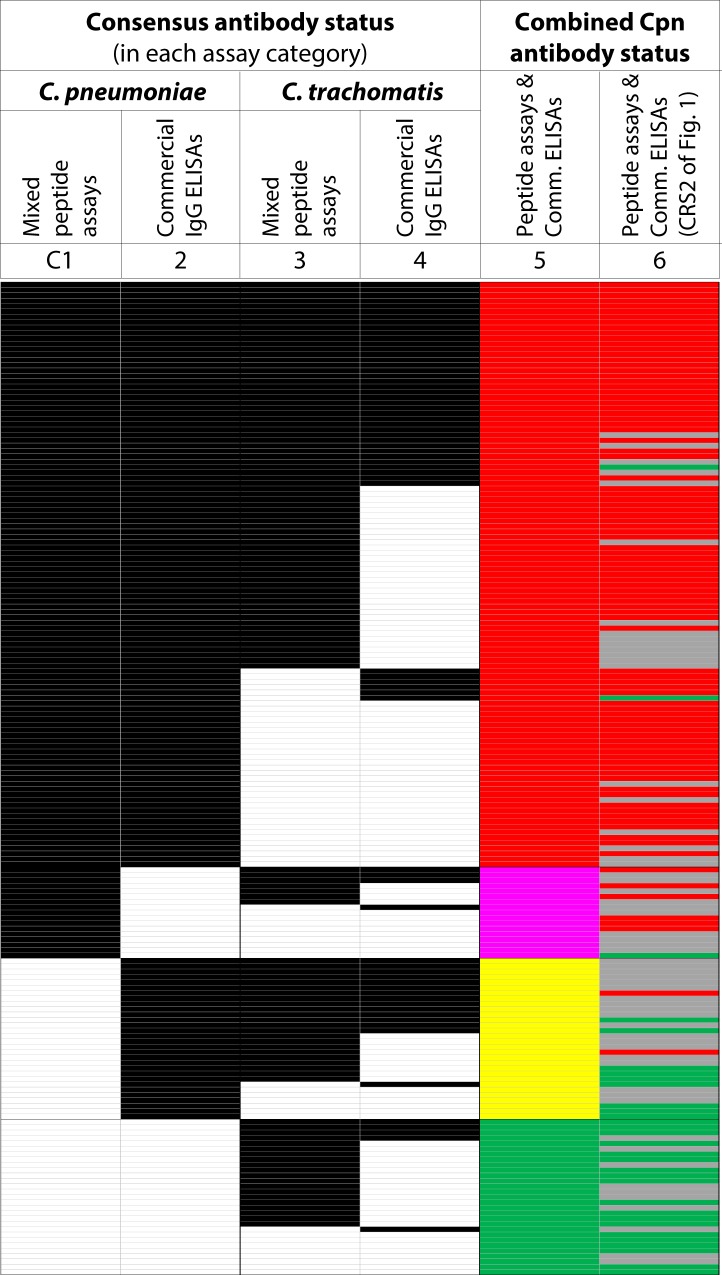
Distribution of anti-*C*. *pneumoniae* and anti-*C*. *trachomatis* antibodies in 185 human sera. To evaluate bias in distribution of anti-*C*. *trachomatis* antibodies among anti-*C*. *pneumoniae* antibody-positive and -negative sera, the 185 blood donor sera were binomially separated by antibody status (Pos or Neg) in Cpn- and Ctr Mix peptide assays and commercial ELISAs. To derive this antibody status (C1-C4), the consensus of 4–6 component assay results for each of the 4 assay categories was used (details in Fig. S5). For this consensus, any serum that was positive in any component assay of a category was considered antibody-positive (black), and antibody-negative if all component assays were negative (white). Column C1 indicates the consensus of 6 mixed peptide anti-*C*. *pneumoniae* antibody assays (Fig. S5). C2 shows the consensus antibody status of the four commercial anti-Cpn IgG ELISAs, with manufacturer defined cutoffs. The consensus antibody status of Ctr mixed peptide assays at previously described cutoffs^47^ is shown in C3, and in C4 the Ctr commercial ELISAs with manufacturer defined cutoffs. In C5, red or green indicates double positive or double-negative sera, respectively, for anti-*C*. *pneumoniae* antibodies in Cpn mixed peptide assays as well as Cpn cELISAs. Single-positive sera in the Cpn mixed peptide assays are shown by pink, and single-positive sera in cELISAs by yellow (C5). The final column (C6) indicates CRS2 status as shown in Figs 1 and S4.

Cross-reactivity for detection of *Chlamydia* species-specific antibodies has been widely reported in serological assays with classical chlamydial antigens (such as EB, EB lysate, OMC, or OmpA, OmcB/Omp2 recombinant proteins) that are used in commercial ELISAs or in the gold standard microimmunofluorescence (MIF) tests^19–27,31–40^. To determine potential cross-reactivities of Cpn and Ctr antigens, we analyzed the distribution of anti-*C*. *trachomatis* and anti-*C*. *pneumoniae* antibody positivity in the 185 human sera for peptide assays and commercial ELISAs (Fig. 2, and see Supplementary Fig. S5). Instead of using a single assay for each test category, we constructed a consensus for anti-*C*. *trachomatis* and anti-*C*. *pneumoniae* antibody status of 4–6 assays each in the two categories of mixed peptide assays and commercial ELISAs (Figs 2, S5). An assays category-dependent distribution bias of anti-*C*. *trachomatis* antibodies among Cpn-positive and -negative sera would identify and quantify potential Ctr-Cpn cross-reactivity.

With Ctr and Cpn mixed peptide assays, Ctr-positive reactivities were 16% lower in Cpn antibody-positive sera than in -negative sera (*P* = 0.187, two-tailed Fisher Exact test; Table 3). With commercial Ctr and Cpn ELISAs, Ctr seroreactivity was 54% higher in Cpn-positive than in Cpn-negative sera in Cpn commercial ELISAs (*P* = 0.005; Table 3). Thus, compared to the mixed peptide assays, commercial ELISAs showed 70% (16% + 54%) increased anti-*C*. *trachomatis* antibody-positivity in anti-*C*. *pneumoniae* antibody-positive over -negative sera (Table 3). This indicates a profound specificity (cross-reactivity) problem of commercial Cpn and Ctr ELISAs, but not of mixed peptide assays. In comparison to seroreactivity against any *Chlamydia* spp. (determined by a *Chlamydia* LPS ELISA; Figs 2, S5, and see Supplementary Table S6), the Cpn commercial ELISAs showed 47% (42% + 5%) excess positive reactivity over the Cpn mixed peptide assays in anti-chlamydial LPS antibody-positive over -negative sera (*P* = 0.005). Thus, differentiation of human anti-*C*. *pneumoniae* from anti-*C*. *trachomatis* or anti-*Chlamydia* spp. antibodies is severely compromised by cross-reactivity of commercial ELISAs, but high specificity is achieved by Cpn and Ctr mixed peptide assays.

**Table 3:** Distribution bias of *C*. *pneumoniae* and *C*. *trachomatis* seroreactivity in 185 human sera.

Comparison between assay types^a^		Cpn antibody status	Ctr antibody status		Ctr antibody distribution			*P* ^e^
Cpn	Ctr		Pos (n)	Neg (n)	Frequency^b^ (% Ctr Pos)	Δ Frequency^c^ (Pos-Neg)	Bias^d^ (Δ Freq/Pos)	
Pept Mix	Pept Mix	Pos = 126	79	47	62.7	−10.2	−0.16	0.187
		Neg = 59	43	16	72.9			
cELISAs	cELISAs	Pos = 139	59	80	42.4	+22.9	+0.54	0.005
		Neg = 46	9	37	19.6			

^a^Consensus antibody status of Cpn and Ctr Pept Mix assays or cELISAs was used (Figs 2, S5).

^b^Distribution of anti-*C*. *trachomatis* antibodies among anti-*C*. *pneumoniae* antibody-positive and -negative sera.

^c^Difference in distribution frequency of anti-*C*. *trachomatis* antibodies among anti-*C*. *pneumoniae* antibody-positive and -negative sera.

^d^Bias towards Ctr-positivity among Cpn-positive sera (excess Ctr positives in Cpn-positive over Cpn-negative sera).

^e^Significance of deviation from random distribution of anti-*C*. *pneumoniae* and anti-*C*. *trachomatis* antibodies is determined by two-tailed Fisher Exact test.

### Cross-reactivities of Cpn and Ctr commercial ELISAs

**Figure 3:**
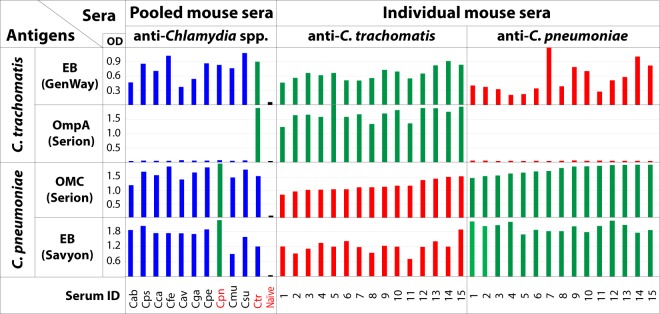
Reactivities of *C*. *pneumoniae* and *C*. *trachomatis* commercial ELISA antigens with anti-*Chlamydia* species-specific mouse sera. The anti-human IgG conjugates of the ELISA kits were substituted by an anti-mouse IgG conjugate. Reactivities of *C*. *pneumoniae* and *C*. *trachomatis* commercial ELISA antigens with homologous antisera that are consistent with expected reactivities are shown by green bars, and cross-reactivities with non-homologous antisera are shown by blue or red bars. Assay backgrounds determined with naïve sera are shown by black bars. Mice were immunized against each of 11 *Chlamydia* spp.^41,45^, and used as individual or pools of individual sera against a single *Chlamydia* spp. Cab indicates a serum pool raised against *C*. *abortus*; Cps, *C*. *psittaci*; Cca, *C*. *caviae*; Cfe, *C*. *felis*; Cav, *C*. *avium*; Cga, *C*. *gallinacea*; Cpe, *C*. *pecorum*; Cpn, *C*. *pneumoniae*; Cmu, *C*. *muridarum*; Csu, *C*. *suis*; and Ctr, *C*. *trachomatis*.

To further evaluate cross-reactivities of Cpn and Ctr commercial ELISA antigens, a panel of *Chlamydia* species-specific pooled and individual mouse antisera were tested with two Cpn and two Ctr commercial ELISAs in which the anti-human IgG conjugate was substituted by an anti-mouse IgG conjugate (Fig. 3). The Savyon (Cpn EB) and Serion (Cpn OMC) ELISA antigens showed very high cross-reactivities with mouse pooled sera raised against each of the 11 *Chlamydia* spp. (Fig. 3). Additional testing of these Cpn ELISAs with individual anti-*C*. *psittaci* and anti-*C*. *trachomatis* sera showed high cross-reactivity, with ~25-fold higher OD over reactivities with naïve mouse sera (Fig. 3). These results conclusively confirm very high cross-reactivity of Cpn commercial ELISA antigens (Savyon and Serion).

In evaluation of potential cross-reactivity of two Ctr commercial ELISAs, the GenWay (Ctr EB) antigen also showed very high cross-reactivities with 15 anti-*C*. *pneumoniae* and 8 anti-*C*. *psittaci* individual sera, similar to the Cpn EB antigen-based Savyon ELISA (Fig. 3). However, the Serion Ctr-specific OmpA fragment antigen did not show cross-reactivity, indicating that this *C*. *trachomatis*-specific antigen captured only anti-*C*. *trachomatis* antibodies.

### High specificity of Cpn and Ctr peptide antigens

To evaluate potential cross-reactivities of Cpn and Ctr peptide antigens with the panel of individual mouse sera raised against *C*. *pneumoniae* and *C*. *trachomatis*, we similarly tested with Cpn and Ctr peptide antigens (see Supplementary Fig. S7). Cpn peptide assays showed strong reactivity with all 15 mouse sera raised against *C*. *pneumoniae* and did not cross-react with any of the 15 mouse sera raised against anti-*C*. *trachomatis* (Fig. S7). Additionally, *C*. *trachomatis*-specific peptide mixture (CtrMix1) reacted with anti-C. *trachomatis* sera but did not react with any of the 15 mouse anti-*C*. *pneumoniae* sera (Fig. S7). Taken together, these results confirm highly specific reactivity of Cpn or Ctr peptide antigens when they are tested either as single antigens or as mixture of multiple peptide antigens.

### Assay sensitivities for detection of anti-*C*. *pneumoniae* IgG in comparison to a peptide antigen-based standard

Given the high cross-reactivities of commercial Cpn ELISAs (Fig. 3 and Table 3), inclusion of these assays in a combined reference standards, such as in CRS2 and CRS3 is inadvisable. To obtain a more reliable standard, CRS4 was derived only from the 13 mixed and 12 individual peptide assays, without the 4 commercial ELISAs (Figs 1, S4). A total of 95 sera with the highest sum score in these 25 peptide assays was considered positive and 40 sera with the lowest score were considered negative (135 sera in total), and the remaining 50 weakly positive borderline sera were excluded.

Using CRS4 for anti-*C*. *pneumoniae* IgG status (see Supplementary Table S8), we evaluated assay sensitivities at 98%, 95%, 90%, 85%, and 80% specificity cutoffs. At 80% specificity cutoff, the top performing mixed peptide assay with Cpn Mix F12 (Table S3) achieved 96% sensitivity, but even at high stringency 98% specificity still achieved 87% sensitivity. The combined result of all 8 top-ranked individual peptide antigens achieved 90-71% sensitivity at these 80–98% specificity cutoffs. In contrast, even the top performing Medac ELISA among the commercial Cpn ELISAs achieved only 71-35% sensitivity, and the poorly performing Euroimmun ELISA only 56-12% sensitivity.

Averaging sensitivity data at the 5 specificity cutoffs, Cpn Mix F12 achieved the highest average sensitivity (93%), followed by Cpn Mix K24 and Cpn Mix J20 (Table 4). Combining the results of the 8 top-ranked individual peptides achieved 82% average sensitivity (Table 4). In contrast, the commercial ELISAs performed significantly lower (56-35% sensitivity) than even the lowest performing peptide assay (*P* ≤ 0.001, two-tailed Fisher exact test, Table 4).

**Table 4:** Sensitivity of anti-*C*. *pneumoniae* IgG detection in comparison to peptide composite reference standard CRS4^a^.

Assay type	Rank #	Antigen	Average sensitivity^b^	AUC^c^
Cpn pooled peptide assays	1	Cpn Mix F12	93	0.976
	2	Cpn Mix K24	91	0.943
	3	Cpn Mix J20	88	0.939
	4	Cpn Mix M48	87	0.916
	5	Cpn Mix G12	86	0.943
Cpn individual peptide assays	1	Average OD of Pept #1–8^d^	82	0.937
	2	Average OD of Pept #1–6	81	0.936
	3	Average OD of Pept #1–4	79	0.927
Cpn ELISAs (commercial)	1	Medac (Proprietary antigen)	56	0.832
	2	Serion (Cpn OMC)	54	0.811
	3	Savyon (Cpn EB)	37	0.768
	4	Euroimmun (Cpn lysate)	35	0.761

^a^CRS4 was the categorical reference in these ROC curve analyses (Figs 1, S4). CRS4 was derived from the 13 mixed and 12 individual peptide assays (without the 4 Cpn commercial ELISAs). A total of 95 sera with the highest combined score in these 25 peptide assays was considered positive, the 40 sera with the lowest score were considered negative (135 sera in total), and the remaining 50 weakly positive borderline sera were excluded in these analyses.

^b^The average assay sensitivity was calculated from the mean of 5 sensitivities obtained for 98%, 95%, 90%, 85% and 80% specificities (Table S8).

^c^AUC, area under the ROC curve.

^d^Derived by averaging seven OD values of 8 separate single peptide assays (8 top-ranked Cpn peptides; Table 2).

### Demographic distribution of anti-*C*. *pneumoniae* and anti-*C*. *trachomatis* antibodies

To evaluate assay performance and the consequences of different cross-reactivity in sero-epidemiology of human chlamydial infection, we analyzed the demographic distribution of anti-*C*. *pneumoniae* and anti-*C*. *trachomatis* antibodies in the 185 human sera, determined by both mixed peptide assays or commercial ELISAs (Figs 2, S5, Table 5). To obtain a reliable status of each serum for anti-*C*. *trachomatis* and anti-*C*. *pneumoniae* antibodies, we used the consensus of 4–6 assays for both peptide assay and commercial ELISA categories as described in Figs 2 and S5. Compared to male donors, female donors had only a 4.9% higher anti-*C*. *pneumoniae* antibody frequency in the Cpn mixed peptide assays (*P* = 0.53, Fisher Exact test), and 7.9% higher in the Cpn commercial ELISAs (*P* = 0.24). However, females had an 18% higher frequency of anti-*C*. *trachomatis* antibodies in the Ctr mixed peptide assays compared to male donors (*P* = 0.013; Table 5), but only 4.5% higher frequency in the Ctr commercial ELISAs (*P* = 0.55). In both mixed peptide assays as well as commercial ELISAs, Caucasian males had lower frequencies anti-*C*. *pneumoniae* and anti-*C*. *trachomatis* antibodies compared to African American and Mixed ethnic origin males (Table 5). Overall, the results in Table 5 show that the commercial Ctr ELISAs underestimated anti-*C*. *trachomatis* antibody prevalence, particularly in female individuals (Figs 2, S5; 46). In contrast, the commercial Cpn ELISAs slightly overestimated the prevalence of anti-*C*. *pneumoniae* antibodies, but frequently misclassified the anti-*C*. *pneumoniae* antibody status (Figs 2, S5, and Table 5).

**Table 5:** Demographics of *C*. *pneumoniae* and *C*. *trachomatis* sero-reactivity^a^.

Cohort (n)	Ethnicity (n)	Anti-*C*. *pneumoniae* antibodies (% pos)^a^				Anti-*C*. *trachomatis* antibodies (% pos)^a^			
		Cpn Mix Pept		Cpn cELISAs		Ctr Mix Pept		Ctr cELISAs	
Female (n = 95)	African American (n = 39)	65.8	**70**.**5**^b^	78.9	**79**.**0**	81.6	**74**.**7**	63.2	**38**.**9**
	Caucasian (n = 20)	60.0		90.0		70.0		15.0	
	Other (n = 37)	81.1		73.0		70.3		27.0	
Male (n = 90)	African American (n = 39)	74.4	**65**.**6**	79.5	**71**.**1**	67.7	**56**.**7**	48.7	**34**.**4**
	Caucasian (n = 15)	46.7		40.0		33.3		13.3	
	Other (n = 36)	63.9		75.0		55.6		27.8	

^a^The consensus antibody status for each assay was calculated by considering a serum as antibody-positive if one component assay of a consensus was positive (47; Figs 2, S5).

^b^Bold numbers indicate cohort average.

## Discussion

In this study, we established simple, yet highly specific and sensitive peptide ELISA methods for detection of anti-*C*. *pneumoniae* antibodies. Assays using mixtures of 12–48 strongly reactive *C*. *pneumoniae*-specific peptide antigens (Cpn Mix F12, K24, J20, M48, or G12) achieved on average 93-86% sensitivity at 80–98% specificity (Tables 4, S8). This result from a single well is better than the 82% sensitivity obtained by combining the results of the individually tested 8 most reactive peptides (Table 4). Highest assay sensitivity is achieved by mixing *C*. *pneumoniae* peptide antigens from several strongly immunodominant proteins rather than multiple antigens from the same protein or from weakly immunodominant proteins (Cpn Mix F12 vs B10; Figs 1, S2, S4, Table 4). High specificity (non-cross reactivity) of the assays is the direct consequence of using peptide antigens that are highly specific for *C*. *pneumoniae*, and not conserved in other *Chlamydia* spp. (usually ≤ 40% sequence identity; Table 1). For most accurate measurement of the anti-*C*. *pneumoniae* antibody status, detection of reactivity to multiple B-cell epitopes in the peptide mixtures mimics reactivity with a complex chlamydial antigen, but with a profound specificity and sensitivity advantage (Tables 4, S8). Thus, these assays simultaneously determine anti-*C*. *pneumoniae* antibodies produced against a wide-spectrum of *C*. *pneumoniae* antigens (Tables 1, S1), including EB outer-membrane structural proteins (OmpA, CrpA, PmpD, Pmp6G/I, Pmp11G/I, Pmp2G/I) as well as inclusion membrane proteins (IncA, IncCT618, IncCT529) and other proteins (YopC, YwbM, PdhC). Importantly, given the severe cross-reactivity problem of current immunoassays (31–40; Table 3 and Fig. 3), these assays fill the void of immunoassays for specific detection of anti-*C*. *pneumoniae* antibodies. For specific and sensitive detection of anti-*C*. *pneumoniae* antibodies, we recommend use of the Cpn Mix F12 peptide antigens that achieved the highest performance (e.g. 91% sensitivity at 95% specificity; Table S8). This antigen mixture for single-well ELISA is composed of 12 highly specific and strongly reactive peptide antigens from 8 immunodominant *C*. *pneumoniae* proteins.

Cross-reactivity between antigens used in detection of anti-chlamydial antibodies has plagued the field since inception^19–27,31–40^. In agreement with these reports, Cpn EB or OMC antigen-based commercial ELISAs exhibited profound cross-reactivity with other *Chlamydia* spp. when tested with mono-specific anti-chlamydial mouse sera (Fig. 3). Similarly, *C*. *trachomatis* EB antigen, but not recombinant *C*. *trachomatis*-specific OmpA antigen, showed cross-reactivity (Fig. 3). In addition, anti-*C*. *pneumoniae* and anti-*C*. *trachomatis* antibodies in the 185 human sera are not randomly distributed when detected by commercial ELISAs. Rather, Cpn antibody-positive sera show a highly significant 54% excess Ctr reactivity over Cpn antibody-negative sera (*P* = 0.005; Table 4). In contrast, anti-*C*. *pneumoniae* and anti-*C*. *trachomatis* antibodies determined by peptide assays are equally distributed (Table 4). These results strongly suggest that the increased Ctr-Cpn co-reactivities in commercial ELISAs are due to the high cross-reactivity of the antigens.

In the absence of any reliable standard for anti-*C*. *pneumoniae* antibody status, we evaluated the Cpn peptide assays by comparison to three composite reference standards (CRS2, CRS3, and CRS4) derived from 25–29 individual assays in 2–3 assay categories. The advantage of such CRS derived from multiple tests is relatively accurate assignment of antibody-negative status. Selection of reliably anti-*C*. *pneumoniae* antibody-negative sera is a critical requirement for assay performance evaluation, given that the ubiquitous nature of human *C*. *pneumoniae* infection has exposed virtually any adult person to *C*. *pneumoniae*. Results of the 29 individual assays (Figs 1, S4) show that almost all donors have been exposed to *C*. *pneumoniae* in their life-time. However, donor sera with low anti-*C*. *pneumoniae* reactivity are classified as antibody-negative for CRS2, CRS3, and CRS4 (Figs 1, S4).

A disadvantage of any CRS is the potential accumulation of false positives if one or more of component tests lack specificity, resulting in poor quality for the positive dataset. To minimize such artifacts, we did not use consensus-based standards CRS2 and CRS3. Rather, we derived CRS2 and CRS3 from the total score of all 29 assays for each serum (Figs 1, S4), by consideration of (i) frequency of positive tests, and (ii) reaction strength. Using CRS2 as reference, Cpn mixed and combined individual peptide assays showed higher assay performance than the 4 commercial Cpn IgG ELISAs (Table 2). The performance of all 29 assays improved when they were referenced against the more reliable CRS3, in which borderline reactive sera from CRS2 were excluded (Figs 1, S4).

Given the high cross-reactivity of commercial Cpn ELISAs with anti-*C*. *trachomatis* antibodies, the commercial Cpn IgG ELISAs rendered CRS2 and CRS3 unreliable for anti-*C*. *pneumoniae* antibody status. Therefore, results of the 4 commercial Cpn ELISAs were not included in a more reliable reference standard, CRS4, which was derived only from 13 mixed and 12 individual peptide assays. Compared to CRS4, the top-performing mixed peptide assays (Cpn Mix F12, K24, J20) achieved 88–84% sensitivity at 98% specificity. In contrast, the 4 commercial ELISAs achieved only 36-12% sensitivity. Acceptable assay specificity for these commercial Cpn ELISAs required a very high OD cutoff to avoid cross-reactivity, while this was not required for the highly specific peptide assay. When the assay cutoff was lowered to 80% specificity, commercial ELISAs achieved 71-56% sensitivities, still 25–40% lower than the 96% sensitivity of Cpn Mix F12 at this cutoff. These results confirm that the severe specificity problem of commercial Cpn ELISAs profoundly compromises sensitivity when the assay cutoff is set for acceptable specificity.

We observed a significantly higher prevalence of IgG antibodies against *C*. *pneumoniae* (83% in CRS2; Figs 1, S4) than against *C*. *trachomatis* (66% in Ctr peptide consensus; *P* = 0.0002, Fisher Exact test; Figs 2, S5). However, serum levels of these IgG antibodies were much higher against *C*. *trachomatis*, particularly in females, than against *C*. *pneumoniae*. In addition, short-lived IgG3 and IgA1&IgA2 antibodies against *C*. *trachomatis* were frequently detected, but not against *C*. *pneumoniae* (Figs 2, S5). Such dominance of short-lived antibody isotypes, indicative of recent *C*. *trachomatis* infection, is a reflection of the endemic and frequent *C*. *trachomatis* infections in the young and sexually active blood donor demographic (mean 22 year-old, range 18–38 years). In contrast, the dominance of highly prevalent, but low level long-lived anti-*C*. *pneumoniae* IgG antibodies suggests infrequent epidemic spread of *C*. *pneumoniae* infection at low-level endemic maintenance.

Ctr peptide assays, but not Cpn peptide assays, correlated with the *Chlamydia* LPS ELISA (Table S6). Given that anti-chlamydial LPS antibodies usually indicate recent chlamydial infection^47^, these results suggest that the *Chlamydia* LPS ELISA detected anti-LPS antibodies that had been elicited mainly by *C*. *trachomatis*, but not by *C*. *pneumoniae*. In contrast, both commercial Ctr and Cpn ELISAs correlated highly significantly with the *Chlamydia* LPS ELISA (Table S6). We attribute this Cpn positivity concomitant with LPS positivity to cross-reactivity of commercial Cpn ELISAs that detected not only anti-*C*. *pneumoniae* antibodies, but also anti-*C*. *trachomatis* antibodies (Fig. 3).

We observed for commercial Cpn ELISAs frequent, mainly false-positive misclassification of anti-*C*. *pneumoniae* antibody status (Figs 2, S5). We attribute this problem to the high cross-reactivity of the complex antigens that are used in commercial Cpn ELISAs. Conversely, we observed many false-negatives for commercial Ctr ELISAs (Figs 2, S5). In the case of OmpA antigen-based Ctr ELISAs, we attribute this to the inherently low-sensitivity of single-epitope ELISAs^46^, because such ELISAs fail to detect antibodies produced against non-OmpA proteins such as Pmps or Incs. In the case of EB-based complex antigens, we attribute the false-negatives to cross-reactivity that forces a high assay cutoff, resulting in low sensitivity. As overall consequence, in our test population of sexually active young adults the performance characteristics of commercial chlamydial ELISAs result in slightly overestimated *C*. *pneumoniae* prevalence due to false-positive misclassifications, and severely underestimated *C*. *trachomatis* prevalence due to frequent false-negatives (Table 5). More broadly, antibody ELISAs using complex antigens of any *Chlamydia* spp. will be relatively specific for the endemic chlamydial species in a given host (i.e. *C*. *trachomatis* in sexually active humans). In turn, the high frequency of antibodies against the most prevalent chlamydial species will strongly compromise antibody detection against low-prevalence *Chlamydia* spp. infection (e.g., *C*. *pneumoniae* or *C*. *psittaci*).

In terms of antigen specificity of anti-chlamydial antibodies, we observed an immunodominance of reticulate body antigens (IncA) for *C*. *pneumoniae*, in contrast to the immundominance of elementary body antigens (OmpA) for *C*. *trachomatis*^46^. This may explain the low sensitivity and cross-reactivity of the EB-based MIF assay for *C*. *pneumoniae* compared to *C*. *trachomatis*^19,20,32^. Thus, inherent *C*. *pneumoniae* antigenic properties (immunodominance of IncA over OmpA, cross-reactivity of complex Cpn antigens) may have prevented accurate *C*. *pneumoniae* serology. Hence, identification of 48 highly reactive peptide antigens from 12 immunodominant *C*. *pneumoniae* proteins is an important discovery that will enable specific detection of anti-*C*. *pneumoniae* antibodies, particularly when used in highly parallel multi-antigen microarray format^52–54^.

With this study, we conclude a series of investigations to establish robust peptide ELISA methodology for *Chlamydia* species-specific human serology. After defining sets of highly, but specifically reactive peptide antigens^41,45^, we have validated assays using individual and mixed peptides for detection of antibodies against both *C*. *pneumoniae* and *C*. *trachomatis*^46,47^. With the use of fully synthetic peptide antigens in simple ELISA formats, these serological assays will now be within reach of any laboratory. Compared to commercially available Cpn and Ctr ELISAs, these peptide assays can provide vastly improved assay sensitivity with unprecedented specificity for simultaneous detection and differentiation of anti-*C*. *pneumoniae* and anti-*C*. *trachomatis* antibodies, and thus improved serodiagnosis of human *Chlamydia* spp. infections.

## Materials and Methods

### *C*. *pneumoniae*-specific peptide antigens

Suitable B cell epitope regions for identification of *C*. *pneumoniae* species-specific peptide antigens had been identified before within polymorphic regions in *Chlamydia* spp. protein alignments^41,45^. These were further subjected to *in silico* B cell epitope identification^42,43^, and a total of 176 *C*. *pneumoniae* species-specific peptide antigens of 20 immunodominant proteins were selected for screening that were highly divergent from other *Chlamydia* spp., but conserved within *C*. *pneumoniae*^41,45^. Peptide antigens were chemically synthesized with N-terminal biotin followed by a serine-glycine-serine-glycine spacer^41^.

### Random peptides as negative controls

For determination of assay background with non-specific peptide antigens, 4 random peptides (23AA long) were generated with the RandSeq tool of ExPASy. A Blast-search confirmed that these AA sequences were not conserved (≤38% sequence identity) with any chlamydial or non-chlamydial protein in the NCBI database.

### *C*. *pneumoniae*-specific peptide antigen mixtures

A total of 13 mixtures of *C*. *pneumoniae* peptide antigens (A5-M48; Fig. S2, Table S3) were prepared. The letter in the alphanumeric CpnMix designation indicates the sequential order and the number indicates the total number of constituent individual peptide antigens.

### Human sera

For selection of anti-*C*. *pneumoniae* antibody-positive and -negative sera (Figs 1, S4), we screened the sera of 95 women and 90 men^47^. The sera originated from healthy blood donors of African American, Caucasian, Hispanic, Asian, or mixed race, and the age of all study subjects ranged from 18–38 years, with an average of 22 years. These sera were collected in the US from blood donors in FDA-licensed and registered collection facilities (Commercial supplier: BioIVT North America & Asia Pacific, Westbury, NY; https://www.bioivt.com/about/quality-assurance/). Sample collection at all donation centers was approved by the Institutional and Review Boards of BioIVT and collaborators. In accordance with the relevant guidelines and regulations, informed consent was obtained and all sera were anonymized.

### Mouse sera

*Chlamydia* monospecies-specific mouse sera for each of the 11 *Chlamydia* spp. were used to confirm specificity of peptide antigens for detection of species-specific antibodies^41,45^. Preparation and pooling of these sera have been described in detail by Rahman *et al*.^41,45^. All animal experimental protocols were approved by the Institutional Animal Care and Use Committee at Auburn University under protocol numbers 2011-1901 and 2014–2468 and performed in accordance with the relevant guidelines and regulations.

### Determination of anti-*C*. *pneumoniae* IgG with four commercial ELISAs

All 185 study sera were tested for anti-*C*. *pneumoniae* IgG with four commercial ELISAs according to manufacturers’ instructions: (i) Savyon *C*. *pneumoniae* elementary body (EB) antigen (Savyon Diagnostics Ltd., Ashdod, Israel), (ii) Serion *C*. *pneumoniae* outer membrane complex (OMC) antigen (Serion Immunologics, Würzburg, Germany), (iii) Euroimmun lysate antigen of cell-culture propagated *C*. *pneumoniae* (Euroimmun AG, Lübeck, Germany), and (iv) Medac chlamydial LPS-free proprietary purified Cpn antigen (Medac GmbH, Wedel, Germany). In addition, study sera were also ELISA-tested for IgG against *Chlamydia* lipopolysaccharide (LPS) by using recombinant chlamydial LPS antigen (Medac GmbH, Wedel, Germany) that detects antibodies against all *Chlamydia* spp. due to genus-wide conservation of chlamydial LPS (Chl LPS).

### Pooling of human sera

Individual sera were first ranked by reactivity with 4 commercial Cpn ELISAs, and 4 serum sub-pools were prepared of (i) 40 high-reactivity male donor sera, (ii) 45 low-reactivity male donor sera, (iii) 45 high-reactivity female donor sera, and (iv) 50 low-reactivity female donor sera.

### Selection of 48 *C*. *pneumoniae*-specific peptide antigens

Out of the 176 tested *C*. *pneumoniae*-specific predicted B-cell epitopes, a total of 48 peptide antigens from 12 immunodominant proteins of *C*. *pneumoniae* were initially selected based on reactivity rank with human serum pools (Table 1). Importantly, the sequences of these *C*. *pneumoniae* peptides are highly evolutionarily divergent from *C*. *trachomatis* (<45% sequence identity; Table 1) and have only a marginal probability (~0.02) of cross-reactivity with antibodies raised against non-*C*. *pneumoniae Chlamydia* spp.^41,43^. In addition, the sequences of these *C*. *pneumoniae* peptide antigens are highly conserved within *C*. *pneumoniae* strains (80–100% sequence identity). Previously, we have determined that identities of 45%, 60%, 75%, and 90% between two peptides translate into 0.02, 0.12, 0.46, and 0.84, respectively, probabilities of antibody cross-reactivity between these peptides^41,43^. Sequences with identities below 40% typically cannot be aligned correctly and the probability of cross-reactivity is less than 1%^41,43^.

### Chemiluminescent and colorimetric ELISAs with *C*. *pneumoniae* peptide antigens

Primary human and mouse antibodies were detected with horseradish peroxidase-conjugated secondary antibodies in ELISAs as described before^41–47^. Polyclonal rabbit anti-mouse or anti-human IgG-h + l cross-adsorbed antibody-HRP conjugates were obtained from Bethyl Laboratories, Inc., Montgomery, TX, USA (Cat# A90-217P and A80-218P). Monoclonal mouse anti-human antibody conjugates were obtained from Southern Biotech, Birmingham, AL, USA: IgG3-HRP (9210-05), IgA1-HRP (B3506B4) and IgA2-HRP (9140-05). For determination of anti-*C*. *pneumoniae* antibody status, all 185 human sera were screened by use of 13 mixed and 12 individual *C*. *pneumoniae* peptide antigens, in addition to the 4 commercial Cpn ELISAs (Figs 1, S4). For cross-reactivity testing of commercial Cpn and Ctr ELISA antigens within *Chlamydia* spp, by use of anti-*Chlamydia* spp. mouse sera, original anti-human IgG conjugates were replaced with polyclonal anti-mouse IgG(h + l) conjugate.

### Composite reference standards (CRS) for anti-*C*. *pneumoniae* antibody status

In the absence of any reliable standard for anti-*C*. *pneumoniae* antibody status, for assay cutoff selection we used CRS1 derived from 29 individual assays (13 mixed and 12 individual Cpn peptide antigen assays, and 4 commercial *C*. *pneumoniae* ELISAs; Figs 1, S4). For chemiluminescence peptide ELISAs, inter-assay coefficient of variation (CV) was approximately ~13%, and intra-assay CV ~9%. For colorimetric peptide ELISAs, inter-assay CV was ~6% and intra-assay CV ~4%. Background-corrected signals (RLU-CV or OD - CV) were used for mixed and individual peptide antigen assays^47^, but OD data for standard ELISAs were processed as described by the manufacturers. The mean OD value of each individual test for the 185 sera was adjusted to the mean OD value of all 29 individual tests. In each assay category, a total of 95 sera was considered antibody-positive based on reactivity rank determined by the sum of the squared OD values of the constituent assays in each category. These 95/185 positive sera matched the average 51.4% anti-*C*. *pneumoniae* antibody prevalence determined by the 4 commercial ELISAs. CRS1 was derived as the consensus of the 3 equally weighted assay categories (Figs 1, S4).

For assay performance evaluation, we used 3 additional composite reference standards (CRS2, CRS3, and CRS4). CRS2 was derived from reactivity scores in all 29 individual assays. Relative to CRS1, an 80.5% specificity cutoff for each assay was chosen for determination of anti-*C*. *pneumoniae* positive and negative sera (Figs 1, S4). Depending on reactivity strength, positive sera were scored from +4, +3, +2, to +1, and negative sera were scored 0. The total score of all 29 individual assays for each serum, shown in the right bar graph in Figs 1 and S4, was derived from the sum of all individual assay scores. For CRS2, a total of 154 sera with the highest combined score in the 29 assays was considered antibody-positive, and 31 sera with the lowest scores were considered antibody-negative. CRS3 was derived from CRS2 after exclusion of 59 weakly-positive borderline sera. CRS4 was derived solely from reactivities in all peptide assays without the 4 commercial ELISAs. The 95 sera with the highest score were considered antibody-positive, 40 sera with the lowest scores were considered antibody-negative, and 50 weakly-positive sera were excluded.

### Consensus of anti-*C*. *pneumoniae* or anti-*C*. *trachomatis* antibody status

For determination of the distribution bias of anti-*C*. *trachomatis* antibodies in dependence of anti-*C*. *pneumoniae* antibodies in the sera, most reliable consensus antibody status in Cpn and Ctr peptide assays and commercial ELISAs were determined. Any serum that was positive in any component assay of a consensus category was considered antibody-positive, and antibody-negative if all component assays were negative (Figs 2, S5). The component assays for these 4 consensus are as follows (Figs 2, S5): (i) 6 Cpn mixed peptide assays with the 4 top-performing Cpn mixes (IgG reactivities of Cpn Mix F12, K24, J20, G12, and IgG3 and IgA reactivities of J20; Table 3); (ii) 4 Cpn commercial IgG ELISAs; (iii) 6 Ctr mixed peptide assays with 2 top-performing Ctr mixes previously reported (IgG, IgG3, and IgA reactivities of CtrMix1 and Ctr Mix2; 47); and (iv) 4 Ctr commercial IgG ELISAs^46^.

### Statistical analyses and receiver operating characteristic (ROC) curves for assay performance

Statistical analyses were performed and graphical outputs generated by the software packages Microsoft Excel 2016 (Microsoft Corporation, Redmond, Washington) or Statistica 7.1 (Statsoft, Tulsa, Oklahoma, USA). ROC curves (Tables 2, 4 and S8) were plotted and area under the curve (AUC) determined as described before^46,47,55^. Sensitivities at 98%, 95%, 90%, 85%, and 80% specificities were calculated from ROC curves. Antibody detection frequencies were compared by two-tailed Fisher exact test.

## Supplementary information

Supplementary Information

## Data Availability

All data of this study are included in this published article and its Supplementary Information File.
