# Supplementary material for: Multi-peptide ELISAs overcome cross-reactivity and inadequate sensitivity of conventional Chlamydia pneumoniae serology
Source: Sci Rep. 2019 Oct 21;9:15078. doi: 10.1038/s41598-019-51501-5 (PMC6803651; doi:10.1038/s41598-019-51501-5)
Supplement: Supplementary file 1 — Supplementary Information [file 41598_2019_51501_MOESM1_ESM.pdf]

Multi-peptide ELISAs overcome cross-reactivity and inadequate sensitivity of conventional *Chlamydia pneumoniae* serology

Kh Shamsur Rahman[1*] and Bernhard Kaltenboeck[1]

[1]Department of Pathobiology, College of Veterinary Medicine, Auburn University, Auburn, AL
[*] Corresponding Author: Kh. Shamsur Rahman (ksr0003@auburn.edu)

Supplementary TABLE S1 Source proteins for *C. pneumoniae*-specific peptide antigens.

Protein	Full name	Cpn locus tag[a]	Ctr locus tag[b]
**IncA/ IncCT119**	Inclusion membrane protein A	CPn0186	CT_119 (IncA)
**Pmp6G/I**	Polymorphic outer membrane protein 6 (Pmp6) G/I family	CPn0444	CT_871 (PmpG) CT_874 (PmpI)
**Pmp21D**	Polymorphic outer membrane protein 21 (Pmp21) D family	CPn0963	CT_812 (PmpD)
**OmpA/ MOMP**	Outer membrane protein A, OmpA (Major outer membrane protein, MOMP)	CPn0695	CT_681 (OmpA)
**YopC/ GspD**	Yop proteins translocation protein C (Type II/III secretion system protein GspD)	CPn0702	CT_674 (YscC)
**CT618/ IncCT618**	Putative inclusion membrane protein	CPn0753	CT_618 (IncCT618)
**Pmp11G/I**	Polymorphic outer membrane protein 11 (Pmp11) G/I family	CPn0451	CT_871 (PmpG) CT_874 (PmpI)
**CT529/ IncCT529**	Putative inclusion membrane protein	CPn0648	CT_529 (IncCT529)
**Pmp2G/I**	Polymorphic outer membrane protein 2 (Pmp2) G/I family	CPn0013	CT_871 (PmpG) CT_874 (PmpI)
**YwbM/ CPn0677**	Hypothetical protein similar to *Bacillus subtilis* YwbM (Lipoprotein for transport of ferrous/ferric iron)	CPn0677	-
**CrpA/ CT442**	15 kDa Cysteine-rich outer membrane protein (Homologous to *C. trachomatis* CT442)	CPn0556	CT_442 (CrpA)
**PdhC**	Pyruvate dehydrogenase complex	CPn0306	CT_247 (PdhC)

[a] 'Cpn locus tag' indicates gene locus tag of *C. pneumoniae* CWL029 genome (NCBI accession: NC_000922.1).

[b] 'Ctr locus tag' indicates gene locus tag of *C. trachomatis* D/UW-3/CX genome (NCBI accession: NC_000117.1).

Peptides # 1-48			Cpn peptide mixes												
#	Protein	AA Position	A5	B10	C10	D12	E11	F12	G12	H10	I10	J20	K24	L30	M48
1	IncA	331-370	■					■	■			■	■	■	■
2		331-345	■									■	■	■	■
3		336-350	■										■	■	■
4		344-363	■										■	■	■
5		361-375	■											■	■
6	Pmp6G/I	0222-0237		■				■			■	■	■	■	■
7		0947-0962		■					■			■	■	■	■
8		0181-0196		■				■			■	■	■	■	■
9		0164-0179		■									■	■	■
10		1223-1238		■							■	■		■	■
11		0899-0914		■										■	■
12		0872-0887		■											■
13		0697-0712		■										■	■
14		1032-1047		■											■
15		0714-0729		■											■
16	Pmp21D	1119-1134			■								■	■	■
17		0140-0155			■						■		■	■	■
18		0640-0655			■					■		■	■		■
19		0191-0206			■						■		■	■	■
20		0147-0186			■			■		■				■	■
21		1131-1170			■			■	■	■		■		■	■
22		0654-0693			■				■	■		■		■	■
23		0717-0732			■									■	■
24		1121-1136			■						■	■			■
25		0521-0536			■				■					■	■
26	OmpA	242-257				■			■				■	■	■
27		158-173				■		■	■	■		■	■	■	■
28		309-324				■			■	■				■	■
29		089-104				■		■	■					■	■
30	YopC	077-092				■		■	■	■		■	■	■	■
31		104-119				■									■
32		196-211				■					■				■
33	CT618	201-216				■		■	■	■		■	■	■	■
34	Pmp11G/I	487-502				■		■			■	■	■	■	■
35		720-735				■							■		■
36		888-903				■		■	■	■		■		■	■
37		334-349				■					■				■
38	CT529	236-275					■				■		■	■	■
39		139-254					■					■	■		■
40	Pmp2G/I	064-079					■	■		■		■	■	■	■
41		024-039					■								■
42	YwbM	265-280					■					■	■		■
43		209-244					■								■
44		233-248					■								■
45	CrpA	173-188					■					■	■		■
46		181-196					■								■
47	PdhC	091-106					■						■		■
48		105-120					■					■			■

Supplementary FIG S2 Composition of *C. pneumoniae* peptide antigen mixes. Each of the black cells indicates that the corresponding peptide antigen numbered in the left column (#1-48 for the 48 *C. pneumoniae*-specific peptides; Table 1) was included in a Cpn peptide mix. The 13 Cpn mixes (A5 to M48) are shown in the top row. The letter in the alphanumeric CpnMix designation indicates the sequential order and the number indicates the total number of constituent individual peptide antigens. CpnMix A to E mixes were composed of 5-12 individual peptide antigens from 1-5 immunodominant proteins of *C. pneumoniae*, and these 5 mixes (A5 to E11) comprised all 48 *C. pneumoniae* peptide antigens. In contrast, another 7 *C. pneumoniae* mixes (F12 to L30) constitute 10-30 peptides chosen from 5-12 *C. pneumoniae* proteins based on the observed individual sero-reactivities with mouse and human sera (Table 1). CpnMix M48 consists of all 48 peptide antigens in a single mixture.

Supplementary TABLE S3 Mouse anti-*C. pneumoniae* IgG detected by *C. pneumoniae*-specific peptide mixes.

Peptide antigen[a]	# of source proteins	# of peptide in the Mix	Constituent peptides	Mouse antiserum (RLU)		
				Cpn	Ctr	Cps
CpnMix A5	1	5	Cpn Pept #1-5	64,000	0	0
CpnMix B10	1	10	Cpn Pept #6-15	0	0	0
CpnMix C10	1	10	Cpn Pept #16-25	23,000	0	0
CpnMix D12	4	12	Cpn Pept #26-37	43,000	0	0
CpnMix E11	5	11	Cpn Pept #38-48	7,000	0	0
CpnMix F12	8	12	Cpn Pept #1, 6, 8, 20, 21, 27, 29, 30, 33, 34, 36, 40	54,000	0	0
CpnMix G12	7	12	Cpn Pept #1, 7, 21, 22, 25-30, 33, 36	51,000	0	0
CpnMix H10	7	10	Cpn Pept #18, 20-22, 27, 28, 30, 33, 36, 40	65,000	0	0
CpnMix I10	5	10	Cpn Pept #6, 8, 10, 17, 19, 24, 32, 34, 37, 38	7,000	0	0
CpnMix J20	12	20	Cpn Pept #1-2, 6-8, 10, 18, 21-22, 24, 27, 30, 33-34, 36, 39-40, 42, 45, 48	33,000	0	0
CpnMix K24	12	24	Cpn Pept #1-4, 6-9, 16-19, 26, 27, 30, 33-35, 38-40, 42, 45,47	28,000	0	0
CpnMix L30	9	30	Cpn Pept #1-11, 13, 16-17, 19-23, 25-30, 33-34, 36, 38, 40	43,000	0	0
CpnMix M48	12	48	Cpn Pept #1-48	27,000	0	0
CtrMix1	9	12	Ctr Pept #1-12 (12 Peptides)	0	136,000	0
CtrMix2	10	12	Ctr Pept #13-24 (12 Peptides)	0	306	0
CpsOmpA	1	1	Cps # 1 (CpsOmpA_333-48)	0	0	125,000
CpsIncA	1	1	Cps # 2 (CpsIncA_321-60)	0	0	135,000
Random Mix	0	0	4 Random peptides (Not conserved in *Chlamydia* or other protein sequences)	0	0	0
Assay diluent	0	0	Coating buffer (no peptide)	0	0	0

[a] Cpn indicates *C. pneumoniae*; Ctr, *C. trachomatis*; Cps, *C. psittaci*. Each of 13 *C. pneumoniae*-specific peptide antigen mixes (CpnMix A5 to M48; Fig. S2), was tested in single microtiter wells. For detection of anti-*C. trachomatis* antibodies, two *C. trachomatis*-specific peptide mixes were tested as previously described (47). For detection of anti-*C. psittaci* antibodies, two *C. psittaci*-specific single peptide antigen of OmpA and IncA proteins were tested (41, 45). For determination of assay backgrounds, each individual serum was tested in wells that were coated with a mixture of 4 random peptides or assay diluent only.

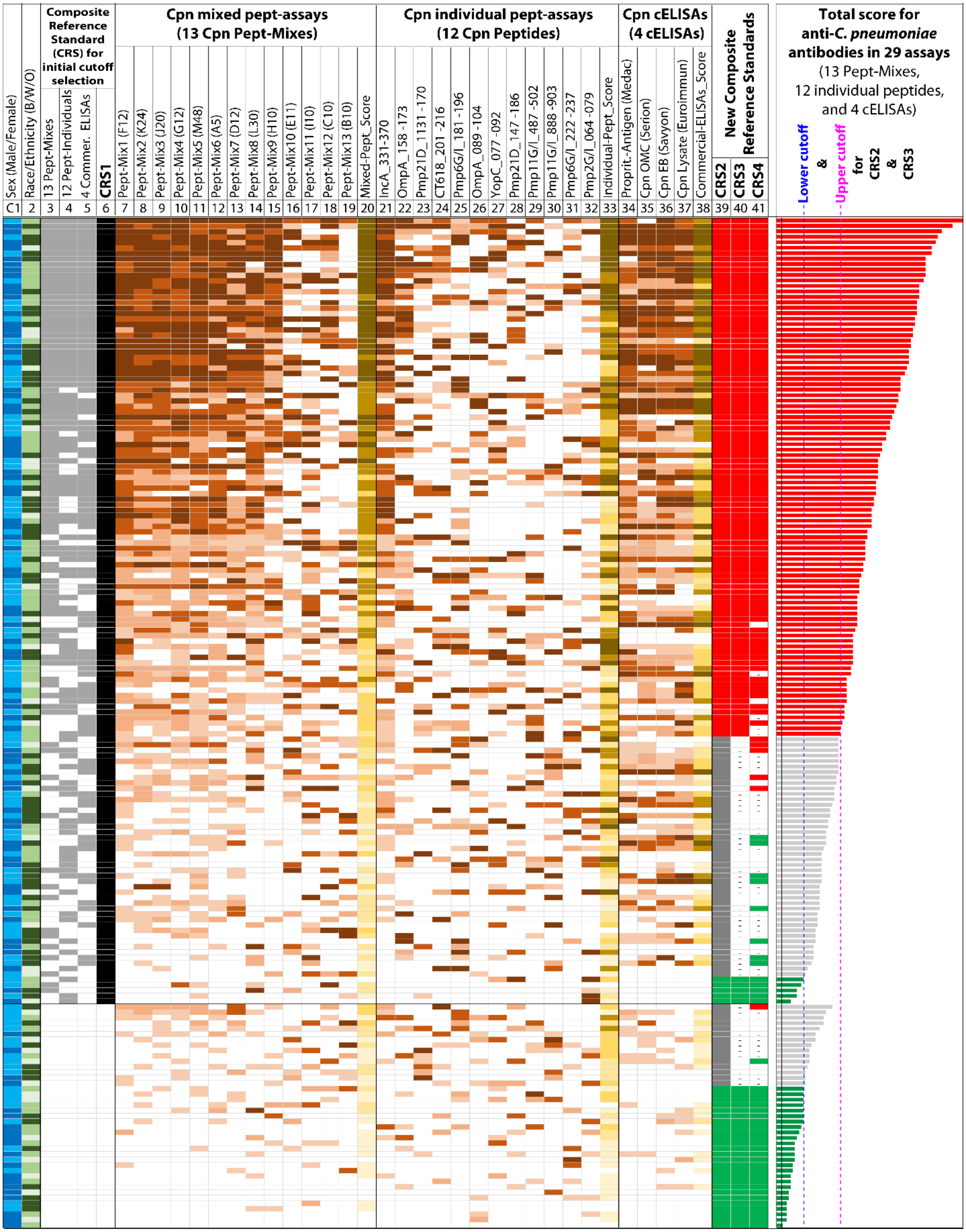

Supplementary FIG S4 Anti-*C. pneumoniae* IgG antibodies detected by 29 assays. To identify sets of anti-*C. pneumoniae* antibody-positive and -negative sera, a panel of 185 blood donor sera

was analyzed with 13 mixed and 12 individual Cpn peptide antigen assays, and 4 commercial *C. pneumoniae* ELISAs. The first column (C1) indicates gender for 95 female (dark-blue) and 90 male donors (light-blue). C2 shows African American (dark-green), Mixed race (green), and White (light-green) ethnic origin of the subjects. Analyses in C3-C5 are input for preliminary composite reference standard 1 (CRS1) in C6. Grey indicates antibody positivity in combined scoring of each assay category. For each serum, the sum of the squared OD values of the individual assays in each category was determined. Cutoff-independently, the 95 top-ranked sera were specified positive, matching the mean 51.4% anti-*C. pneumoniae* IgG prevalence determined in the commercial ELISAs. Next, assays were ranked semi-quantitatively by cutoff-dependent quartile score for positivity or negativity. Color intensity in C7-19, C21-32, and C34-37 indicates positive scoring from +4, +3, +2, to +1, and white cells indicate negative sera (score = 0). The 80.5% specificity cutoff for each assay in C7-19, C21-32, and C34-37 was based on CRS1. Assay category scores in C20, C33, and C38 were derived from the sum of component individual scores. The total score of all 29 individual assays for each serum, shown in the right bar graph, was derived from the sum of all individual assay scores. For CRS2 (C39), a total of 154 sera with the highest combined score in the 29 assays was considered antibody-positive (red + grey), and 31 sera with the lowest scores were considered antibody-negative (green). CRS3 (C40) was derived from CRS2 (C39) after exclusion of 59 weakly-positive borderline sera (grey). CRS4 (C41) was constructed as peptide assay-based standard from the combined scores of 13 mixed and 12 individual peptide assays (without the 4 commercial ELISAs). The 95 sera with the highest score were considered antibody-positive (red), 40 sera with the lowest scores were considered antibody-negative (green), and 50 weakly-positive sera were excluded. CRS2, CRS3, and CRS4 were used in subsequent performance evaluations.

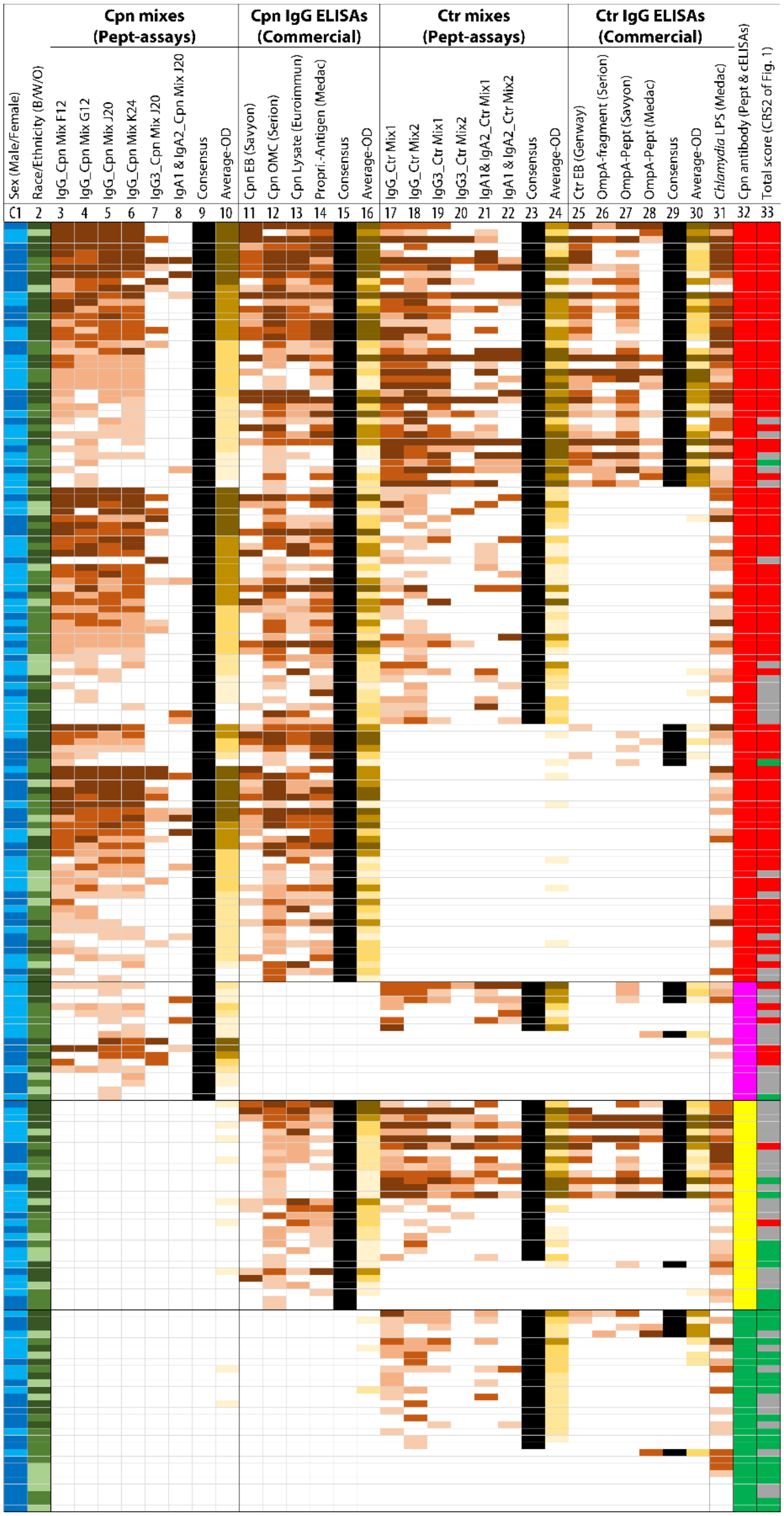

Supplementary FIG S5 Distribution of anti-*C. pneumoniae* and anti-*C. trachomatis* antibodies in 185 human sera. To evaluate bias in distribution of anti-*C. trachomatis* antibodies among anti-

C. pneumoniae antibody-positive and -negative sera, results of the panel of 185 blood donor sera for Cpn and Ctr Mix peptide assays and commercial ELISAs are displayed by assay categories. Columns C1 and C2 indicate donor gender and ethnicity (Fig. 1, Fig. S4). Columns C3-C8 indicate anti-*C. pneumoniae* IgG, IgG3, and IgA reactivities with Cpn Mixes F12, G12, K24, and J20 (Fig. 1, Fig. S4). For each of the four IgG assays, the 95 highest reactive sera were considered positive, and quartile reaction intensities are displayed as described for Fig. 1 and Fig S4. C11-C16 show antibody status of the four commercial anti-Cpn IgG ELISAs, with manufacturer defined cutoffs for determination of antibody-positive and -negative sera. Ctr mixed peptide assays are shown in C17-C24 at previously described cutoffs (47), and Ctr commercial ELISAs with manufacturer defined cutoffs are shown on C25-C30. For reliable determination of binomial antibody status (Pos or Neg), 4-6 component assays in the 4 assay categories were used to derive the consensus of anti-*C. pneumoniae* and anti-*C. trachomatis* antibodies (C9, C15, C23, and C29). Columns C10, C16, C24, and C30 show the average reactivity of each serum in each category. For this consensus, any serum that was positive in any component assay of a category was considered antibody-positive (black), and antibody-negative if all component assays were negative (white). C31 indicates results of the commercial Medac anti-*Chlamydia* LPS IgG ELISA. Column C32 combines the C9 and C15 anti-*C. pneumoniae* antibody status of the sera. Double positive or double-negative sera for anti-*C. pneumoniae* antibodies in Cpn mixed peptide assays as well as Cpn cELISAs are shown by red and green, respectively. Single-positive sera in the Cpn mixed peptide assays are shown by pink, and single-positive sera in cELISAs by yellow (C32). The final column (C33) indicates CRS2 status as shown in Fig. 1 and Fig S4.

Supplementary TABLE S6 Distribution bias of *C. pneumoniae* and *C. trachomatis* seroreactivity in *Chlamydia* LPS-positive and -negative sera.

**Comparison between (Assay type)**[a]		**Cpn/Ctr antibody status**	**LPS antibody status**		**LPS antibody distribution**			***P***[e]
**Cpn/Ctr**	**LPS**		**Pos** (n=112)	**Neg** (n=73)	**Frequency**[b] **(% LPS Pos)**	**Δ Frequency**[c] **(Pos-Neg)**	**Bias**[d] **(Δ Freq/Pos)**	
Cpn Pept Mix	Chl LPS	Cpn Pos = 126	75	51	59.5	- 3.2	- 0.05	0.748
		Cpn Neg = 59	37	22	62.7			
Cpn cELISAs		Cpn Pos = 139	94	45	67.6	+ 28.5	+ 0.42	<0.001
		Cpn Neg = 46	18	28	39.1			
Ctr Pept Mix	Chl LPS	Ctr Pos = 122	87	35	71.3	+ 31.6	+ 0.44	<0.001
		Ctr Neg = 63	25	38	39.7			
Ctr cELISAs		Ctr Pos = 68	59	9	86.8	+ 41.5	+ 0.48	<0.001
		Ctr Neg = 117	53	64	45.3			

[a] Consensus Cpn and Ctr antibody status of Pept Mix assays or cELISAs were used (Fig. 2, Fig. S5). Due to genus-wide conservation of chlamydial LPS (Chl LPS), the Medac ELISA using recombinant LPS antigen detects antibodies against any *Chlamydia* spp.

[b] Distribution of anti-LPS antibodies among anti-*C. pneumoniae* or anti-*C. trachomatis* antibody-positive and -negative sera.

[c] Difference in distribution frequency of anti-LPS antibody-positive sera among anti-*C. pneumoniae* or anti-*C. trachomatis* antibody-positive and -negative sera.

[d] Bias towards co-positivity of anti-LPS antibody and anti-*C. pneumoniae* or anti-*C. trachomatis* antibody (excess Cpn/Ctr-positives in LPS-positive over LPS-negative sera).

[e] Significance of deviation from random distribution of anti-LPS antibody-positive sera and Cpn/Ctr-antibody among is determined by two-tailed Fisher Exact test.

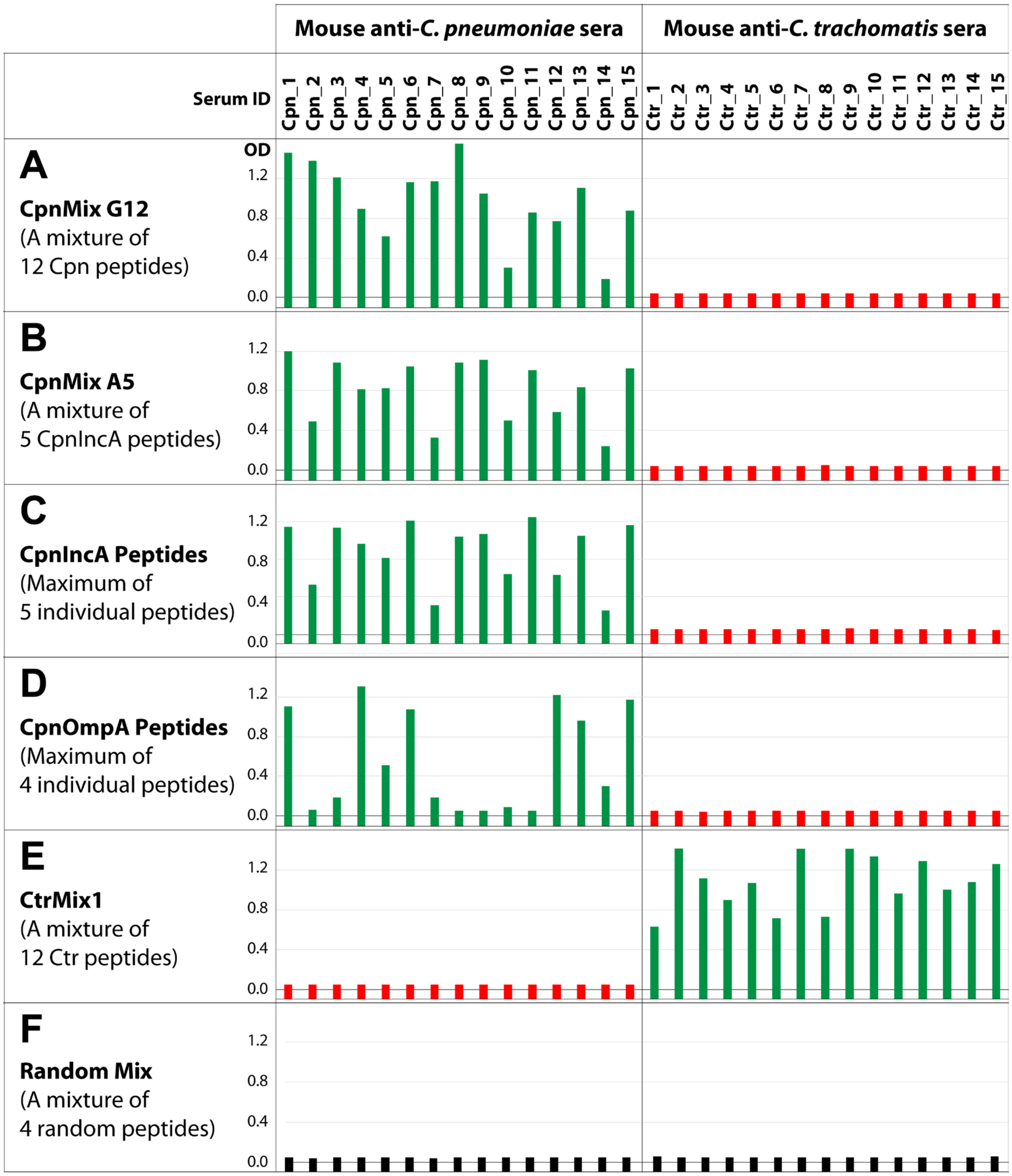

Supplementary FIG S7 Reactivities of Cpn and Ctr peptide antigens with anti-*C. pneumoniae* and anti-*C. trachomatis* mouse sera. Reactivity as expected (green bars), cross-reactivity (red bars), and assay backgrounds (black bars) of *C. pneumoniae* and *C. trachomatis* peptide antigens are shown. (A) Sero-reactivity of Cpn Mix G12 with pooled sera from a group of mice that was immunized intranasally 3× with live *C. pneumoniae* or *C. trachomatis* bacteria (41, 45). (B) Sero-reactivity of Cpn Mix A5. (C) Strongest reactivity among the 5 *C. pneumoniae* IncA peptides of Cpn Mix A5 (Table 1). (D) Strongest reactivity among the 4 *C. pneumoniae* OmpA peptides (Table 1). (E) Sero-reactivity of Ctr Mix1 for anti-*C. trachomatis* antibodies (47). (F) Reactivity of a mixture of 4 random peptides for determination of assay background.

Supplementary TABLE S8 Sensitivity of anti-*C. pneumoniae* IgG detection evaluated by CRS4 at different specificity cutoffs[a].

Assay type	Antigen	Sensitivity at specificity of[b]				
		**98%**	**95%**	**90%**	**85%**	**80%**
**Cpn pooled peptide assays**	Cpn Mix F12	87	91	94	96	96
	Cpn Mix K24	88	90	91	92	93
	Cpn Mix J20	84	87	89	90	91
	Cpn Mix M48	84	86	88	89	89
	Cpn Mix G12	77	83	87	90	91
**Cpn individual peptide assays**	Average OD of Pept #1-8[c]	71	79	84	88	90
	Average OD of Pept #1-6	69	77	84	87	90
	Average OD of Pept #1-4	65	74	81	85	88
**Cpn ELISAs (commercial)**	Medac (Proprietary antigen)	35	47	59	66	71
	Serion (Cpn OMC)	36	47	57	64	69
	Savyon (Cpn EB)	13	25	39	49	57
	Euroimmun (Cpn lysate)	12	24	37	47	56

[a] CRS4 was the categorical reference in these ROC curve analyses (Fig. 1, Fig. S4). CRS4 was derived from the 13 mixed and 12 individual peptide assays (without the 4 Cpn commercial ELISAs). A total of 95 sera with the highest combined score in these 25 peptide assays was considered positive, the 40 sera with the lowest score were considered negative (135 sera in total), and the remaining 50 weakly positive borderline sera were excluded in these analyses.

[b] Assay sensitivity was calculated at 98%, 95%, 90%, 85% and 80% specificities.

[c] Derived by averaging seven OD values of 8 separate single peptide assays (8 top-ranked Cpn peptides; Table 2).
